# Co-Infection Dynamics of a Major Food-Borne Zoonotic Pathogen in Chicken

**DOI:** 10.1371/journal.ppat.0030175

**Published:** 2007-11-16

**Authors:** Beate Skånseng, Pål Trosvik, Monika Zimonja, Gro Johnsen, Lotte Bjerrum, Karl Pedersen, Nina Wallin, Knut Rudi

**Affiliations:** 1 MATFORSK, Norwegian Food Research Institute, Ås, Norway; 2 Department of Chemistry, Biotechnology and Food Science, Norwegian University of Life Sciences, Ås, Norway; 3 Centre for Ecological and Evolutionary Synthesis, University of Oslo, Oslo, Norway; 4 Department of Informatics, University of Oslo, Oslo, Norway; 5 National Veterinary Institute, Oslo, Norway; 6 IVAR, Stavanger, Norway; 7 National Veterinary Institute, Technical University of Denmark, Århus N, Denmark; 8 Department of Applied Microbiology, Lund University, Lund, Sweden; 9 Hedmark University College, Hamar, Norway; Tufts University School of Medicine, United States of America

## Abstract

A major bottleneck in understanding zoonotic pathogens has been the analysis of pathogen co-infection dynamics. We have addressed this challenge using a novel direct sequencing approach for pathogen quantification in mixed infections. The major zoonotic food-borne pathogen *Campylobacter jejuni,* with an important reservoir in the gastrointestinal (GI) tract of chickens, was used as a model. We investigated the co-colonisation dynamics of seven C. jejuni strains in a chicken GI infection trial. The seven strains were isolated from an epidemiological study showing multiple strain infections at the farm level. We analysed time-series data, following the *Campylobacter* colonisation, as well as the dominant background flora of chickens. Data were collected from the infection at day 16 until the last sampling point at day 36. Chickens with two different background floras were studied, mature (treated with Broilact, which is a product consisting of bacteria from the intestinal flora of healthy hens) and spontaneous. The two treatments resulted in completely different background floras, yet similar *Campylobacter* colonisation patterns were detected in both groups. This suggests that it is the chicken host and not the background flora that is important in determining the *Campylobacter* colonisation pattern. Our results showed that mainly two of the seven C. jejuni strains dominated the *Campylobacter* flora in the chickens, with a shift of the dominating strain during the infection period. We propose a model in which multiple C. jejuni strains can colonise a single host, with the dominant strains being replaced as a consequence of strain-specific immune responses. This model represents a new understanding of C. jejuni epidemiology, with future implications for the development of novel intervention strategies.

## Author Summary

Pathogenic bacteria that can be transferred from animals to humans represent a highly potent human health hazard. Understanding the ecology of these pathogens in the animal host is of fundamental importance. A major analytical challenge, however, is the fact that individual animal hosts can be colonised by multiple strains of a given pathogen. We have addressed this challenge by developing a novel high-throughput approach for analyses of mixed strain infections. We chose Campylobacter jejuni colonisation of the chicken gastrointestinal (GI) tract as a model. C. jejuni is a major cause of food-borne disease in humans, and chickens are considered a main reservoir from which this bacterium may enter the food chain. We analysed the co-colonisation of seven C. jejuni strains in two groups of chickens with very different background GI microfloras. We found that mainly two of the C. jejuni strains colonised the chickens, with a shift in the dominant coloniser during the infection period. The C. jejuni colonisation pattern, however, was little affected by the dominating GI microflora. We propose a model where the chicken immune response is the important determinant for C. jejuni colonisation, and suggest that multiple strain colonisation could be a way of maintaining stable infections in the animal host. This new knowledge is very important for future development of novel intervention strategies to prevent C. jejuni from entering the human food chain.

## Introduction

Understanding the ecology of zoonotic pathogens in the animal host is crucial for controlling infections in humans [1,2]. Our knowledge is limited, however, with respect to within-host dynamics of pathogens. One reason for this is the lack of experimental models addressing the effect of co-infections on pathogen colonisation. Here, we present the application of a novel approach in which we can quantify mixed populations directly in infected material using direct sequencing and statistical analysis, without prior cultivation of bacterial isolates [3]. The aim of our work was to determine strain dependence and dynamics in a Campylobacter jejuni co-infection model in two different background floras, mature (treated with Broilact, a product consisting of bacteria from the intestinal flora of healthy hens) and spontaneous, using the direct sequencing approach.

C. jejuni is a leading cause of diarrhoeal disease and food-borne gastroenteritis in humans. This bacterium is zoonotic and poultry is considered a major reservoir for transmission to humans [4]. C. jejuni is able to colonise the GI tract of chickens without causing any disease in the host [5,6]. The principal localisation of C. jejuni is the lower gastrointestinal tract, especially the caecum [7]. Multiple C. jejuni genotypes have been found in the GI tracts of individual chickens and within commercial broiler flocks [8–13]. It has also previously been shown, using antibiotic-resistant strains, that there can be interference in colonisation between pairs of C. jejuni strains [14]. The effects of co-infection dynamics and multiple strain infections, however, have not yet been described. This knowledge is important for our understanding of the epidemiology of *Campylobacter*, and for the development of intervention strategies that can prevent C. jejuni from entering the food chain.

The seven isolates selected in our study were isolated from an epidemiological field experiment [15]. We have both field data and experimental infection data for the strains used, and we present evidence for a relatively rapid shift in the dominating C. jejuni strain between the ages of 27 and 30 days in our infection trials. We also show that this shift is relatively unaffected by the dominating microflora. The relevance of our findings is discussed in the context of the epidemiology and control of C. jejuni.

## Results

### Field Data

We found C. jejuni–positive chickens in three out of four farms using real-time PCR quantification. The positive flocks, at farms A, C, and D, were the same as those found in the study by Johnsen et al. [15]. The selected flocks at farms A and D became infected at 2 wk of age, while the flock at farm C became infected at 3 wk of age [15]. The number of C. jejuni–positive samples measured with real-time PCR was 53% at farm A, 38% at farm C, and 18% at farm D. The colonisation level relative to the total flora for the C. jejuni–positive chickens from farms A, C, and D were −3.89 log, −4.11 log, and −3.92 log (with standard deviations of 0.85, 0.85, and 0.67), respectively (Figure S1).

Six of the C. jejuni–positive caecum samples were chosen for studying the diversity of C. jejuni isolates in the chicken caecum (three chickens from each of the farms A and D). From both farms, samples were selected to represent high, medium, and low C. jejuni colonisation levels. Products from the amplification of the housekeeping genes *gltA* (citrate synthetase; *n* = 62), *glnA* (glutamine synthetase; *n* = 80), *glyA* (serine hydroxymethyltransferase; *n* = 3), and *tkt* (transketolase; *n* = 1) were cloned and sequenced. Most polymorphic sites were found in the genes of *gltA* and *glnA*. The alignments showed that there were multiple genotypes present at the same time in all six chickens, and this was particularly the case for farm A, the chicken from which contained up to nine different genotypes of both *gltA* and *glnA* genes.

A total of 127 C. jejuni strains from the field study [15] were screened with regard to two of the partial multilocus sequencing (MLST) housekeeping genes, *gltA* and *glnA*. We found a larger variety of genotypes of the *gltA* gene than of the *glnA* gene. However, comparison of the partial MLST with the amplified fragment length polymorphism (AFLP) typing by Johnsen et al. [15] showed good agreement with both the *gltA* and the *glnA* genotypes (Figure S2). We therefore chose the *gltA* gene for further analyses, as it had the highest degree of variation.

**Figure 1:**
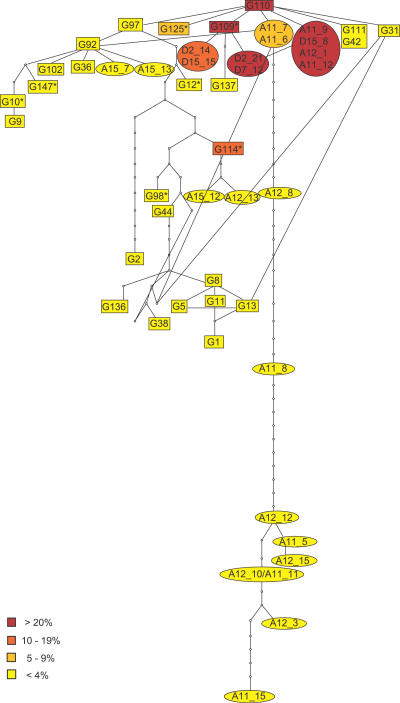
Estimation of Gene Genealogies of the Sequences of the *gltA* Gene Detected in This Study (One Sequence per Genotype), Using TCS Software [17] Squares represent sequences from strains from the field study, while sequences from the cloned samples are shown in ovals. Proportions of the sequence type, based on the sequence group they belong to (strain or clone), are marked in colours. The maximum number of steps connecting two haplotypes is indicated. Strain G110 emerged as the haplotype with the highest out-group probability. Strains used in the infection model are marked with an asterisk (*).

The gene genealogies were estimated using the TCS software v1.21 based on statistical parsimony [16,17]. Sequences representing each of the genotypes detected in the cloned samples and from the strains from the field study were used in the analysis (Figure 1). Strain G110 (not used in the infection model) came out as the haplotype with the highest out-group probability. It is particularly interesting to note that we can see a separate grouping of sequences from the cloned samples from farm A. A BLAST search showed an approximately 90% similarity to the *gltA* gene of C. jejuni, which was the first hit on the result table. These unique sequences were not found in any of the strains from the field study.

### Experimental Infection of C. jejuni in Chickens

Seven C. jejuni strains were selected for use in the infection model; these strains were G10, G12, G98, G109, G114, G125, and G147 (information about these strains are given in Table 1, and marked in Figure 1). The selection was based on specific mutations in the *gltA* gene, differences in the AFLP pattern, and the fact that strains from all farms were represented in the infection model.

**Table 1:**
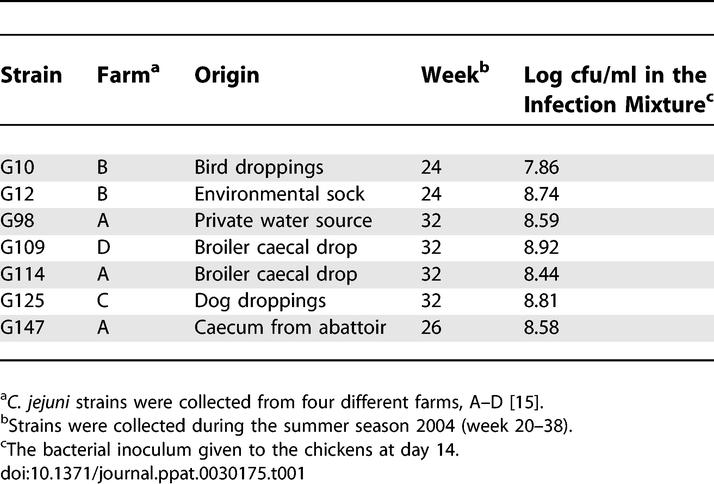
C. jejuni Strains Used in the Infection Model

**Figure 2:**
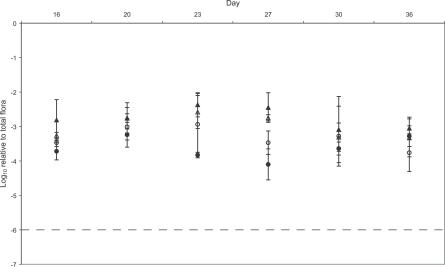
Colonisation of C. jejuni during the Experiment from Day 16 until Day 36 Colonisation of C. jejuni relative to the total flora in caecum, measured by real-time PCR. The chickens were infected with C. jejuni at day 14. Triangles (Δ and ▴) represent the chickens with spontaneous background flora (housed in two separate isolators). Circles (○ and •), represent chickens treated with Broilact (housed in two separate isolators). The grey bars indicate the standard deviation of the counts, and the dashed line indicates the detection limit.

All chickens were infected with a dose of approximately 8.7 log_10_ colony-forming units (cfu) of the C. jejuni mixture. This is within the range expected when chickens eat infected faecal material since intestinal contents often harbour 5–9 log_10_ cfu per gram [18]. Colonised chickens appeared healthy and showed no signs of disease. Plate counts showed a caecal colonisation level of approximately 8 log_10_ cfu g^−1^ caecum during the infection period (results not shown). The C. jejuni–specific quantitative real-time PCR amplification [19] gave an average colonisation level between −2 and −4 log values relative to the total flora in the caecum for the infected chickens (Figure 2).

Analysis of variance (ANOVA) was performed on data from the real-time quantification using Minitab v14.2. The factor variables treatment and day (day 16 to 36) were tested against the colonisation level as response variable. The results showed a significant effect of treatment on the colonisation levels (*p*-value < 0.001). The mean colonisation levels of C. jejuni in Broilact-treated chickens were lower than the mean colonisation levels of C. jejuni in chickens with spontaneous background flora. This difference in colonisation level of C. jejuni between the two different groups is most evident at day 27 (Figure 2).

### Typing of C. jejuni Isolates Colonising Chickens

**Figure 3:**
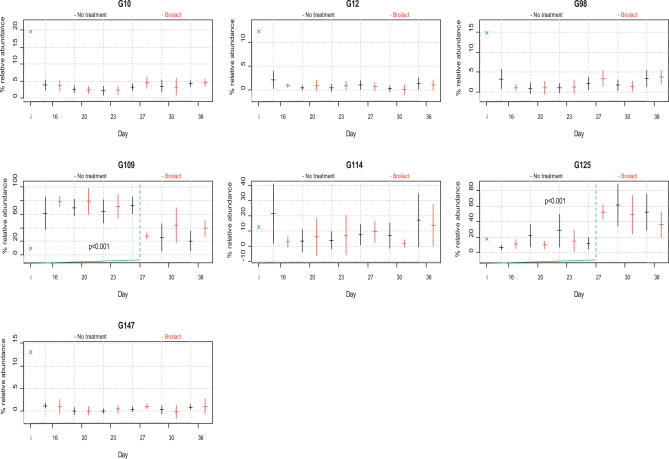
Relative Abundances of the Seven C. jejuni Isolates in Broilact- and Non-Treated Chickens Sampled at Six Consecutive Time Points The green crosses indexed as “i” on the abscissae are relative abundances in the inoculate used to infect the chickens. Horizontal lines are mean relative abundances within groups of treated (red) or non-treated (black) chickens sampled on the days indicated on the abscissae. Vertical black and red lines are two times the standard error within each group. In the plots for isolates G109 and G125, horizontal dotted green lines indicate the day on which a shift in dominant colonising isolate is observed. The *p*-values presented in these plots were computed from F-tests comparing the full models (with time trends and interaction terms) with the null models. The data for the non-treated group for day 16, 20, and 23 are from Trosvik et al. [3].

We found that mainly two of the seven C. jejuni isolates colonised the caecum, isolates G109 and G125. Other strains only colonised sporadically (Figure 3), but at levels above the detection limit (*p* < 0.05). In order to identify time trends and effects of Broilact treatment on G109 and G125 relative abundances, ANOVA was carried out. Since our response variables were proportions, the logit transform (i.e., log-odds) was applied to the estimated relative abundances prior to modelling. Day of sampling was found to have a significant effect on both G109 and G125 abundances (*p* < 0.001 in both cases), indicating a pronounced time trend. The analysis did not detect any significant effect of Broilact treatment, except as an interaction term with day of sampling (*p*-values of 0.006 and 0.003 for G109 and G125, respectively), indicating a significant influence of Broilact treatment on the time trend. Further inspection of the models showed that the interaction terms were significant only for day 27 in both cases (*p*-values of 0.003 and 0.049 for G109 and G125, respectively). Figure 3 shows an evident shift in abundances of the two isolates in Broilact-treated chickens sampled on day 27, with a precipitous decline in G109 proportions and a corresponding upsurge of G125. The shift persists until the end of the sampling period. The same shift can be observed for non-treated chickens, but not until day 30.

To further investigate the observed time trends, the logit transformed proportions were modelled as continuous functions of time. Both for G109 and G125, models were fitted separately for Broilact- and non-treated chickens (Figure S3). For G109, significant negative time trends were found within both the treated and non-treated groups (*p* < 0.001 in both cases). For G125, equally significant positive trends were found. This suggests constant change rates in relative abundance through time, but in opposite directions, for the two isolates. When treatment was included in the model as an interaction term, we did not find significant differences in constant change rates in Broilact- and non-treated chickens for either C. jejuni isolate.

The direct sequencing method was also applied to duodenum and jejunum samples. The colonisation levels in these parts of the intestinal tract are low. The typing information obtained, however, showed the same colonisation trend as that of the caecum samples (results not shown).

The minimum spanning network in Figure 1 shows that the main colonisers, G109 and G125, are both connected near the haplotype with the highest out-group probability, G110. G109 is a sister group to dominating sequences from cloned samples, and this isolate belongs to a group of dominating sequences. This indicates that the isolate could be quite common in the environment of chickens. The other isolates used in the infection model were more distinct from the proposed out-group.

### Classification of the Total Flora

**Figure 4:**
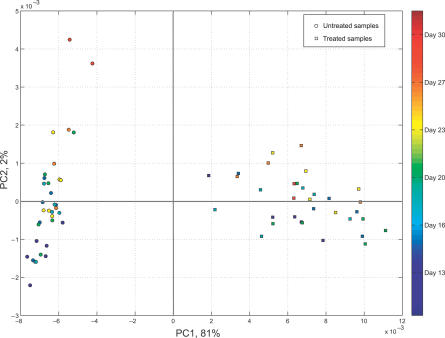
PCA Scores Plot of the Classification of the Total Flora The colouring of samples represents the age of the chicken when the samples were collected. The percentage shown on each axis represents the portion of the total variance explained by that principal component. Untreated samples are represented by the chickens with spontaneous microflora, while the treated samples are the chickens given Broilact.

A direct sequencing approach of a universally conserved region of the 16S rRNA genes was used for the classification of the total caecal flora [3]. Principal component analysis (PCA) was performed on the mixed spectra. The two first principal components (PCs) explained 83% of the total variance in the dataset (Figure 4). Adding more factors did not markedly increase the percentage explained of variation. The first PC, explaining 81% of the total variance, clearly separated samples collected from Broilact-treated and untreated chickens as determined by multiple linear regression (MLR) (*p* < 0.001).

The signature sequence 5′-CAG ACG GCC TTT TAA GTC ANC TCT GAA AGT TTG CGG GTC AAC CGT AAA ATT-3′ (corresponding to Escherichia coli position 581 to 631) deduced form the positive loading for PC1 (Figure S4) showed that the Broilact-treated chickens were associated with bacteria belonging to *Bacteroidetes*, while the negative loading signature sequence 5′-TAN ACG GGA NAA GCN NGN CTG GAN TGA AA ACC CNG GGC TCA ACC CCG GGA CGT GCT TGT G-3′ (E. coli position 581 to 637) showed that the untreated chickens were associated with *Clostridia*.

The second PC, explaining 2% of the total variance in the data, correlated well with the time of sampling (*p* < 0.001 by MLR). The untreated chickens had a better separation on the second PC than the Broilact-treated chickens. The loading plot for this component showed that *Clostridia* was associated with early colonisation (signature 5′-TAG AGT GCN GGN GNG GTA NGA GGT T-3′, E. coli position 652 to 675), while *Gammaproteobacteria* (signature 5′-AGA AGA GTA AAA NNC AAN AT-3′, *E. coli* position 661 to 675) were associated with later time points.

## Discussion

In this study we demonstrated a shift in the dominating *C. jejuni* strains colonising chickens during the course of an infection. This shift was found to be independent of individual variation among chickens. Furthermore, we detected up to nine genotypes in a single chicken at the farm level. We have also shown that there was no major effect of the dominating microflora on the C. jejuni colonisation pattern.

*Plasmodium* is, to our knowledge, the only pathogen that has been investigated in detail with respect to mixed strain/species infections [20]. A model proposed for *Plasmodium* is that a specific density-dependent adaptive host immune response prevents the outgrowth and suppresses the dominant strain/species. This response also suppresses the non-dominant strains/species in a non-specific manner. When the negative-selected dominant *Plasmodium* population drops below a certain threshold, then the density-dependent reaction is turned off, allowing the outgrowth of a new population that is not controlled by a specific immune response [21].

The colonisation pattern for C. jejuni determined by our confection trials can be explained using the *Plasmodium* model. It is also well known that C. jejuni has a variable surface antigen structure [22], analogous to that of *Plasmodium* [23]. Variable surface antigen structures are an adaptation to avoid the host adaptive immune response. We propose that infections with multiple C. jejuni strains in nature allow for stable infections in a host with an adaptive immune response, such as chickens. Multiple strain infections could in fact be a general mechanism among pathogens to maintain stable infections. C. jejuni is a prokaryote pathogen with a main reservoir in the gut, whereas *Plasmodium* is a eukaryote blood parasite. Thus, it is likely that other pathogens in other reservoirs have developed similar mechanisms.

The Broilact treatment had a major effect on the total microflora, while there was only a minor effect on C. jejuni colonisation. The Broilact effect on C. jejuni may not be directly linked to the microflora itself, but could rather be an indirect effect of the dominating microflora on the chicken host immune system. It is likely that the intestinal microflora derived from Broilact represents a lower burden on the host than the spontaneous microflora. This is supported by the findings that the spontaneous microflora was dominated by *Clostridia*, whereas the microflora derived from Broilact-treated chickens was dominated by *Bacteroidetes*. It is well known that *Clostridia* contain bacteria that are a burden to the host, while most bacteria belonging to *Bacteroidetes* are beneficial [24]. The immune systems in the Broilact group of chickens may therefore be more responsive than those in spontaneous group. This would explain both the lower colonisation levels and the advanced occurrence of shifts in the dominating C. jejuni strain. The reason why we do not favour an explanation related to a direct effect of the dominating microflora is the fact that C. jejuni infection courses are similar under completely different background flora regimes. It could of course be argued that it is the mucosal microflora and not the dominant microflora that is important in determining the C. jejuni colonisation pattern. This explanation, however, would require a rapid shift in the mucosal microflora corresponding to the C. jejuni shift. We find this unlikely. Thus, the most parsimonious explanation is that the chicken host is the most important factor in determining the C. jejuni colonisation pattern, and not the mucosal nor the dominating luminal microflora.

There have been numerous trials using Broilact or other competitive exclusion (CE) approaches to combat C. jejuni [25–27]. The general conclusion from these trials is that it is possible to reduce, but not eliminate, C. jejuni by CE. The issue of CE, however, has not yet been properly addressed with respect to the host immune system, making it difficult to separate the potential CE effect from the host immune response. In particular, it would be interesting to know if CE approaches targeting mucosal surfaces also trigger the adaptive and/or innate immune system, and if the host responses are confounded with the proposed CE effects [13,26,28].

Until now technology has limited pathogen infection models to single or double-strain infections. Our seven-strain infection model points towards a novel understanding of the epidemiology of C. jejuni, which again could lead to a new way of thinking with respect to the development of intervention strategies. Certainly, investigating multiple strain infections will also be important for understanding the epidemiology of other bacterial pathogens, and for learning how to combat them.

## Materials and Methods

### Field study.

Johnsen et al. [15] investigated four Norwegian broiler farms (farms A–D) for genetic diversity of *Campylobacter* in broilers and in the environment of broiler farms. These farms had a history of producing *Campylobacter*-positive broiler flocks, and samples were taken from 11 May to 14 September 2004. The farms were visited nine times, three times prior to the broiler flock sampling, weekly during the 4-wk growing period of the selected flocks, and twice thereafter. During the growing period of the broiler flocks, broiler caecal material and five different sites inside the broiler house were sampled.

A total of 144 Campylobacter spp. strains obtained from the sampling were typed using AFLP [15]. Of the 144 Campylobacter spp. strains, 127 were identified as C. jejuni. Seven of the C. jejuni strains were selected for use in the infection model (described in “Experimental infections,” below). Thirty whole caeca were sampled from each flock (farms A–D) at slaughter, and samples were frozen at −80 °C for further quantification and typing of colonising C. jejuni.

### Experimental infections.

The experimental infections were carried out at Foulum Research Centre (Tjele, Denmark) following Danish legislation for animal welfare and use of experimental animals. The chickens used for the experimental infections were conventional broiler chickens (Ross 308) of mixed sex, purchased at 1-d-old from a local hatchery (DanHatch). The C. jejuni–free animals were transferred directly from the hatchery to the experimental unit, where they were housed in isolators (Montair Andersen B.V. HM 1500). The four experimental groups were kept in separate isolators, and group size was 23–24 chickens, each 1 d of age. Two of the groups were treated with Broilact (Orion Oyj) at day 1, while the two other experimental groups did not receive any microbial treatment. All four experimental groups were inoculated with a mixture of seven different C. jejuni strains at day 14. The chickens were inoculated individually by crop instillation with 500 μl of the bacterial suspension (approximately 9 log_10_ cfu/ml), using a 1-ml syringe with an attached flexible tube.

Bacterial inoculum was prepared from cultures on blood agar base plates (Oxoid) supplemented with 5% (v/v) calf blood (BA) and incubated at 42 °C for 48 h under microaerobic conditions. Bacterial suspensions were prepared by shaking of bacterial material in 0.9% saline at 4 °C. For each strain, the bacterial suspension was adjusted to an optical density of approximately OD_620_ = 0.6. According to the measured OD_620_, the strains were mixed in equal concentrations. Cfu for the bacterial suspensions of each strain were determined (Table 1), and total cfu of the mixture were determined before and after inoculation, and the minimal colonisation dose was calculated as the mean of these counts.

During the experiments, three chickens were removed from each group (a total of six chickens per treatment) twice a week after the inoculation until day 36. The chickens were killed by decapitation, and each chicken was sampled and examined individually. Contents from caecum, duodenum, and jejunum were collected separately in tubes and stored at −80 °C. The separation of the small intestine into jejunum and ileum is often done at the Meckel's diverticulum, which is the site where the yolk sack is attached. This definition has been used in this experiment.

### Growth-dependent C. jejuni quantification.

C. jejuni counts were determined as cfu per gram of chicken caecum. The caecal contents were weighed and diluted in buffered peptone water. Ten-fold dilution series were made and streaked onto modified charcoal cefoperazone deoxycholate agar (mCCDA) plates (CM 739, Oxoid) with selective supplement (SR 155, Oxoid). The plates were incubated microaerobically at 42 °C for 48 h.

### DNA isolation, purification, and quantitative real-time PCR amplification.

DNA isolation and purification of contents from caecum, duodenum, and jejunum were performed using an automated procedure as described earlier by Skånseng et al. [19]. Quantification of C. jejuni was performed relative to the total flora [19]. Universal 16S rDNA primers and probe [29] were used for quantification of the total flora. C. jejuni–specific real-time PCR was performed using the primer and probe set described by Nogva et al. [30].

Using AmpliTaq Gold DNA polymerase (Applied Biosystems) in the real-time PCR reaction, the mixture contained 1× TaqMan Buffer A (Applied Biosystems), 5 mM MgCl_2_, and 200 μM dNTP mix. Universal 16S rDNA PCR reactions contained 0.2 μM of each primer, 0.1 μM probe, 1.25 U AmpliTaq Gold DNA polymerase, and 1 μl DNA in a 25-μl PCR reaction. C. jejuni–specific PCR reactions contained 0.3 μM of each primer, 0.02 μM probe, 2.5 U AmpliTaq Gold DNA polymerase, and 4 μl DNA in a 50-μl reaction. With the use of DyNAzyme II Hot Start DNA Polymerase (Finnzymes Oy) in the real-time PCR, the reaction mixture contained 1× Hot Start Buffer (Finnzymes), 0.5 μM ROX reference dye (Invitrogen), and 200 μM dNTP mix. Universal 16S rDNA real-time PCR contained 0.2 μM of each primer, 0.1 μM probe, 1 U DyNAzyme II Hot Start DNA Polymerase, and 0.5 μl DNA in a 25-μl PCR reaction. C. jejuni–specific real-time PCR reactions contained 0.3 μM of each primer, 0.02 μM probe, 1 U DyNAzyme II Hot Start DNA Polymerase, and 2 μl DNA in a 25-μl reaction.

The amplification profile was 40 cycles of 95 °C for 30 s and 60 °C for 1 min, with an initial heating step of 95 °C (AmpliTaq Gold) or 94 °C (DyNAzyme II Hot Start) for 10 min. The reactions were performed in an ABI PRISM 7900 HT Sequence Detection System (Applied Biosystems) and the data were analysed using the SDS 2.2 Software (Applied Biosystems).

### Multilocus sequence typing (MLST).

Amplification of the C. jejuni housekeeping genes, *glnA*, *gltA*, *glyA*, and *tkt* was performed using primers [31] listed in Table S1. The PCR amplification reactions contained 1× PCR Buffer II (Applied Biosystems), 5 mM MgCl_2_, 200 μM dNTP mix, 0.2% BSA (New England Biolabs), 2.5 U AmpliTaq Gold DNA polymerase (Applied Biosystems), 0.2 μM of each primer, and 5 μl DNA in a 25-μl reaction. The amplification profile was an initial step of 95 °C for 10 min, then 40 cycles of 95 °C for 30 s, 50 °C for 2 min, and 72 °C for 30 s, and a final extension at 72 °C for 7 min.

PCR products were cloned into a plasmid vector using a TOPO TA Cloning Kit (Invitrogen) as previously described by Rudi et al. [32]. Cells with insertions were amplified using primers HU (5′-CGC CAG GGT TTT CCC AGT CAC GAC G-3′) and HR (5′-GCT TCC GGC TCG TAT GTT GTG TGG-3′). The PCR products were purified before sequencing. This was done by adding 10 U of Exonuclease I and 2 U of Shrimp Alkaline Phosphatase (USB Corporation) to 8 μl of PCR product. The thermal profile was 37 °C for 15 min and 80 °C for 15 min. The sequencing reaction contained 0.75× BigDye v1.1/3.1 Sequencing Buffer, 1 μl BigDye Terminator v3.1 Cycle Sequencing Kit, 0.32 μM of primer M13, and 3 μl of purified PCR product in a 10-μl reaction. The sequencing reaction was carried out in 25 cycles of 96 °C for 15 s, 50 °C for 10 s, and 60 °C for 4 min. Purification of the sequence products was performed with the Montage SEQ_96_ Sequencing Reaction Cleanup Kit (Millipore) using a Biomek 2000 Workstation (Beckman Coulter). Ten microliters of Hi-Di Formamide (Applied Biosystems) was added to the purified sequence products. Sequencing was performed on an ABI PRISM 3100 Genetic Analyzer (Applied Biosystems).

### Direct sequencing for typing of *C. jejuni.*

*Typing of C. jejuni isolates for use in the infection model.* Amplification of the partial C. jejuni housekeeping genes *glnA* and *gltA* (Table S1) was performed on isolates from the study of Johnsen et al. [15]. The PCR amplification reactions contained 1× PCR Buffer II (Applied Biosystems), 5 mM MgCl_2_, 200 μM dNTP mix, 2.5 U AmpliTaq Gold DNA polymerase (Applied Biosystems), 0.2 μM of each primer, and 1 μl DNA in a 25-μl reaction. The amplification profile was an initial step of 95 °C for 10 min, then 30–40 cycles of 95 °C for 30 s, 50 °C for 2 min, and 72 °C for 30 s, and a final extension at 72 °C for 7 min. Sequencing was performed using 0.5 μM of primers gln1F or glt1F. Further sequencing was performed as described in the section “Multilocus sequence typing (MLST)”.

*Typing of C. jejuni from the infection model*. Amplification of the *C. jejuni gltA* genes in the caecum samples from the experimental infection was performed using glt1F and glt1R [31] (Table 1). The PCR amplification reactions contained 1× Hot Start Buffer (Finnzymes), 200 μM dNTP mix, 1U DyNAzyme II Hot Start DNA Polymerase (Finnzymes), 0.2 μM of each primer, and 1 μl DNA in a 25-μl reaction. The amplification profile was an initial step of 95 °C for 10 min, then 35 cycles of 95 °C for 30 s, 50 °C for 2 min, and 72 °C for 30 s, and a final extension at 72 °C for 7 min.

The PCR products were purified before sequencing (as described in “*Typing of C. jejuni isolates for use in the infection model*”). The sequencing reaction contained 0.75× BigDye v1.1/3.1 Sequencing Buffer, 2 μl BigDye Terminator v3.1 Cycle Sequencing Kit, 0.25 μM of primer glt1F, and 3 μl of purified PCR product in a 20-μl reaction. The sequencing reactions were carried out in 25 cycles of 96 °C for 15 s, 50 °C for 10 s, and 60 °C for 4 min.

Precipitation of the sequencing products was performed using an ethanol/EDTA- procedure (Applied Biosystems). Five microliters of 125 mM EDTA and 60 μl of 96% ethanol were added to the sequencing products and the reactions were mixed by inverting the 96-well plate four times. The products were incubated at room temperature for 15 min before centrifuging at 4,500 rpm for 45 min at 4 °C. The supernatants were then removed, and the inverted plate was spun up to 1,000 rpm. Sixty microliters of 70% ethanol were added to the products and the mixtures were centrifuged at 4,500 rpm for 30 min at 4 °C. The supernatants were removed by inverting the plate, and the plate was spun at 1,000 rpm for 1 min. The pellets were resuspended in 14 μl of Hi-Di Formamide. Sequencing was performed on an ABI PRISM 3100 Genetic Analyzer.

*Quantification of C. jejuni isolates colonising chickens*. For estimating relative strain abundances in the caecal samples, we used MLR analysis of mixed sequence electropherograms according to the linear mixture model [3]. Briefly, this entails modelling the DNA sequence spectrum from a mixture of homologous gene fragments as a linear combination of the pure sequence spectra constituting the mixture. In our case, there were seven strains of C. jejuni, giving us the following model: **y**_j_ = β_j1_ **x**_1_ + β_j2_ **x**_2_ + β_j3_ **x**_3_ + β_j4_ **x**_4_ + β_j5_ **x**_5_ + β_j6_ **x**_6_ + β_j7_ **x**_7_ + **ɛ**_j_, where y_j_ is a mixed spectrum, x_1_,...,x_7_ are the pure strain spectra, β_1_,...,β_7_ are regression coefficients, and **ɛ**_j_ is an error term. According to the linear mixture model, the regression coefficients may be interpreted as relative amounts of the strain corresponding to spectrum **x**_i_ (i = 1,...,7) [33], if the system is additive. Additivity was tested by applying the model to a test set, and no serious deviations from a linear relationship between response and covariates were found.

For the analysis we used spectral data from 14 SNPs in the *gltA* gene, extracting spectral readings at the point of base calling, as well as three flanking readings on each side. The resulting (98×4) spectral matrices were re-scaled by multiplying all values within blocks of (7×4) (i.e., emission readings for one polymorphic site) with the ratio between the total spectral mean and the block mean. The matrices were subsequently unfolded and mean normalised [3].

### Direct sequencing for classification of the total microflora.

DNA isolated from caecum was amplified with universal 16S rDNA primers [29]. The PCR mixture contained 0.2 μM of each primer, 1 U DyNAzyme II Hot Start DNA Polymerase, 1× Hot Start Buffer, 200 μM dNTP mix, and 1.0 μl DNA in a 25-μl PCR reaction. The amplification profile was an initial step of 94 °C for 10 min, then 30 cycles of 94 °C for 30 s, 60 °C for 30 s, and 72 °C for 30 s, and a final extension at 72 °C for 7 min.

The PCR products were purified before sequencing, using 0.4 μl of ExoSap-IT (USB Corporation) to 5 μl of PCR product. Thermal profile was 37 °C for 30 min and 80 °C for 15 min. The sequencing was performed using a universally conserved primer U515F [34] with C-tail extension (U515Fc30), consisting of 30 bases on the 5′-end. The sequencing reaction contained 0.75× BigDye v1.1/3.1 Sequencing Buffer, 1 μl BigDye Terminator v1.1 Cycle Sequencing Kit, 0.32 μM of primer U515Fc30, and 0.5 μl of purified PCR product in a 10-μl reaction. The sequence reaction was performed by 25 cycles of 96 °C for 15 s and 60 °C for 4 min. Precipitation of the sequence products was performed using BigDye XTerminator Purification Kit (Applied Biosystems), according to instructions supplied by the manufacturer. Sequencing was performed on an ABI PRISM 3100 Genetic Analyzer (Applied Biosystems).

The most appropriate spectral region in 16S rRNA gene for data analysis was found to be between the conserved start sequence 5′-ATTTANTGGGT-3′ and end sequence 5′-GAATTCNNNGTGTA-3′, covering a region corresponding to nucleotide positions 565 to 677 in the E. coli 16S rRNA gene. We found this region to comprise enough DNA sequence dissimilarity to distinguish main groups of bacterial DNA sequences found in the mixtures. The trimmed DNA sequence spectra were imported into Unscrambler software v9.6 (CAMO Software) and analysed using PCA [35]. PCA is used to separate essential information from noise in data with many variables and thus allows viewing the analysed data as easily interpretable plots. The scores separate the samples analysed, while the loading plot shows which parts of the sequence spectra are important for the separation of the samples. The correlation analyses were done using MLR based on the orthogonal PCA scores. The significance of the correlations was determined using ANOVA. These analyses were done using the Unscrambler software. We used empirically determined signature sequences identified in the loading plot to identify bacterial that differ in the samples analysed. The signature sequences were subsequently assigned to a hierarchical taxonomy using Probe Match in the Ribosomal Database Project II (http://rdp.cme.msu.edu/).

## Supporting Information

Figure S1 *C. jejuni*–Positive Chickens (Δ) from the Infected Farms in the Field Study The mean colonisation levels of C. jejuni at the different farms are marked with a black line (—).
(1.0 MB EPS)

Figure S2 Dendrogram Based on AFLP Fragment Patterns of 144 Campylobacter spp. Strains from the Field Study by Johnsen et al. [15] This figure shows only the C. jejuni strains. The types of the *gltA* and *glnA* genes for the C. jejuni strains are given in the two columns at the right side of the figure. (ND, not detected)
(3.0 MB TIF)

Figure S3 Logit Relative Abundances of Isolates G109 and G125 Modelled as Continuous Functions of Day of Sampling In the figure, models have been fitted separately for Broilact- and non-treated chickens. Both strains show highly significant constant change rates for both strains regardless of treatment. In the plot legends “a” is the model intercept, “b” is the slope, and “p” gives the model's significance level.
(1.8 MB EPS)

Figure S4 Mixed Sequence Spectra (a) The mean DNA spectrum of all 16S rRNA gene mixture samples is presented together with nucleotide position information according to 16S rRNA gene sequence of E. coli. (b) The first loading shows which nucleotides or regions in 16S rRNA gene discriminate between the non-treated and the treated samples. The positive peaks correspond to the nucleotides that are found in 16S rRNA gene mixtures extracted from treated samples, while the negative peaks are related to the nucleotides found in 16S rRNA gene mixtures of non-treated samples. (c) The second loading provides information about which part of the DNA sequence spectra that differ with respect to the age of the chickens.
(5.0 MB EPS)

Table S1 PCR Primers Used to Amplify Partial C. jejuni Housekeeping Genes (*glnA*, *gltA*, *glyA*, *tkt*) [31] (29 KB DOC)

### Accession Numbers

The housekeeping genes *gltA*, *glnA*, *glyA*, and *tkt* from the six caecum samples studied from the field study were sequenced and deposited in GenBank (http://www.ncbi.nlm.nih.gov/Genbank/index.html) under accession numbers EF546072, EF546074, EF546076–EF546138, EF546140, EF546151–EF546155, EF546157–EF546169, EF546171–EF546185, EF546187–EF546230, EF546232, EF546233, and EF546235.

## Abbreviations

AFLP: amplified fragment length polymorphism
ANOVA: analysis of variance
CE: competitive exclusion
cfu: colony-forming units
GI: gastrointestinal
*glnA*: glutamine synthetase
*gltA*: citrate synthetase
*glyA*: serine hydroxymethyltransferase
MLR: multiple linear regression
MLST: multilocus sequencing
PC: principal component
PCA: principal component analysis
*tkt*: transketolase
