# Supplementary material for: Co-Infection Dynamics of a Major Food-Borne Zoonotic Pathogen in Chicken
Source: PLoS Pathog. 2007 Nov 16;3(11):e175. doi: 10.1371/journal.ppat.0030175 (PMC2077904; doi:10.1371/journal.ppat.0030175)
Supplement: Supplementary file 1 [file ppat.0030175.st001.doc]

**Supplementary Table 1.** PCR primers used to amplify partial *C. jejuni* housekeeping genes (*glnA, gltA, glyA, tkt*) [31]

| **PCR primers** | **Sequence (5’ - 3’)** |
| --- | --- |
| gln1F | AAG GAC AAA TGT ATG AAA AAT GTC CAA GAA |
| gln1R | GTT TGA ACC ATT TCA GAR CGA ATA TCT AC |
| glt1F | CGT CTT TTT GAT TCT TTY CCT GAT AAT |
| glt1R | GAA GTW GAA GCA TTT TGY TCA TGA TCT G |
| gly1F | GGA CAT TTA ACW CAT GGT GCA AAA GTA AG |
| gly1R | GAA TTA ATT TTT TTA GCA AGC TCT TCA TCA T |
| tkt1F | TTA AAA ATT TCC GYC AAC TTC AT |
| tkt1R | GYG MYT CAA AAC GCA TTT TTA CAT |
